# Bacterial Meningitis Associated With Spontaneous Bacterial Peritonitis Caused by Escherichia coli in a Patient With Alcoholic Liver Cirrhosis

**DOI:** 10.7759/cureus.77937

**Published:** 2025-01-24

**Authors:** Daichi Umemoto, Hiroaki Nishioka

**Affiliations:** 1 Department of General Internal Medicine, Kobe City Medical Center General Hospital, Kobe, JPN

**Keywords:** adult bacterial meningitis, alcoholic liver cirrhosis, disturbed consciousness, escherichia coli, spontaneous bacterial peritonitis

## Abstract

Spontaneous *Escherichia coli *meningitis in adults is uncommon and many cases coexist with urinary tract infections. We herein report a 66-year-old man with *E. coli* meningitis complicated by spontaneous bacterial peritonitis. A patient with liver cirrhosis admitted with disturbed consciousness was diagnosed with spontaneous bacterial peritonitis due to *E. coli* and administered ceftriaxone. His level of consciousness improved, but it worsened on day 4. A cerebrospinal fluid examination revealed *E. coli* meningitis. On day 10, the patient died due to hepatorenal syndrome. Thus, spontaneous *E. coli* meningitis can coexist with spontaneous bacterial peritonitis, which may lead to a delayed diagnosis.

## Introduction

Bacterial meningitis is a life-threatening neurological emergency that requires early diagnosis and immediate antimicrobial therapy. The most common cause of bacterial meningitis in adults is *Streptococcus pneumoniae* and *Neisseria meningitidis*, along with *Listeria monocytogenes* in people over 50 years of age [1]. Although *Escherichia coli *is a common cause of neonatal meningitis, it is an uncommon cause of this condition in adults. Most cases of *E. coli* meningitis in adults have been reported to occur after head trauma or neurosurgery, and spontaneous *E. coli *meningitis in the absence of a known risk factor is rare [2,3]. *E. coli* causes meningitis via direct infection or hematogenous seeding from a distant site of infection. While urinary tract infection is the most frequently reported distant lesion in *E. coli* meningitis [4,5], spontaneous bacterial peritonitis has rarely been reported.

Alcoholic liver disease is the most common chronic liver disease worldwide. It includes fatty changes, steatohepatitis, fibrosis, and eventually cirrhosis. Most individuals consuming >40 g of alcohol per day develop alcoholic fatty liver. Diagnosis of alcoholic liver disease involves assessing patients for alcohol use disorder and signs of advanced liver disease. The degree of alcoholic fatty liver and liver fibrosis can be determined by image examinations, such as ultrasonography, computed tomography, transient elastography, magnetic resonance imaging, and transient elastography, measurement of serum biomarkers, and liver biopsy histology. Patients with cirrhosis are at risk of experiencing various complications, which can result in a significant reduction in their life expectancy.

This report presents a case of spontaneous *E. coli* meningitis associated with spontaneous bacterial peritonitis in a patient with uncompensated alcoholic liver cirrhosis.

## Case presentation

**Figure 1:**
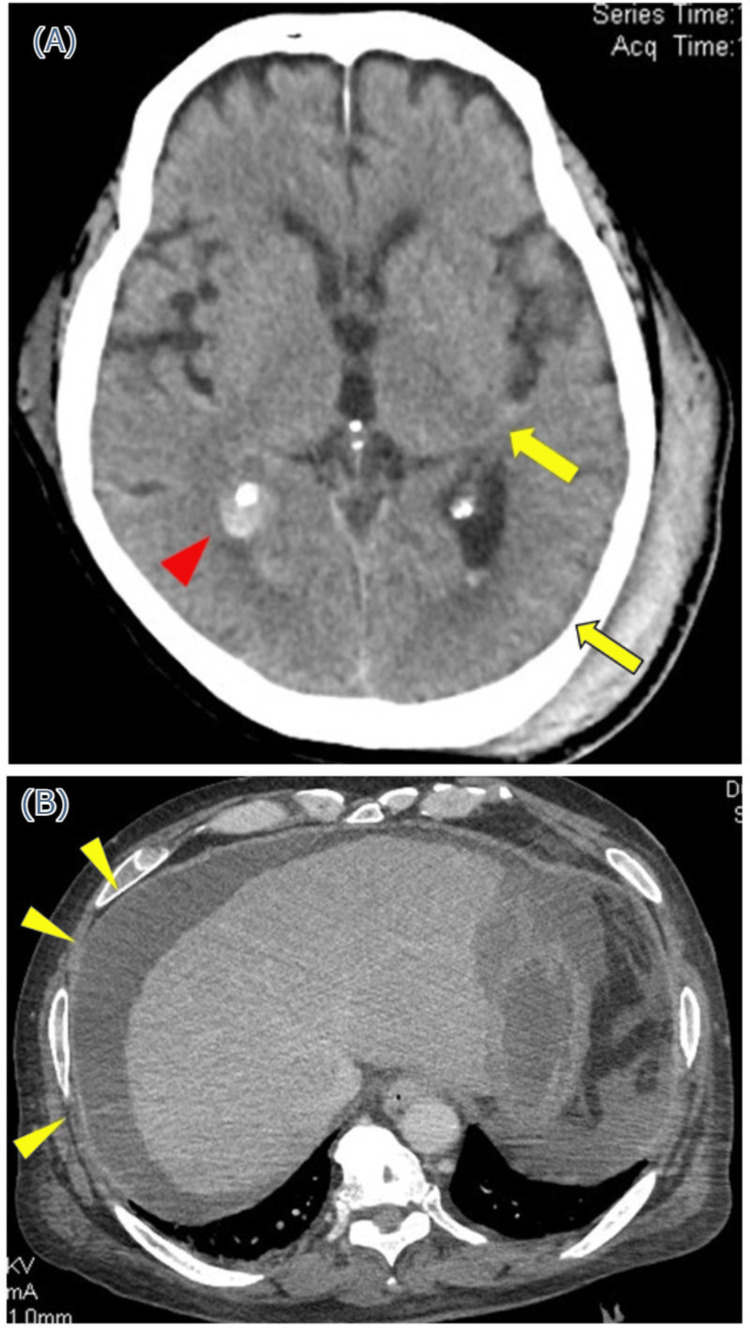
Computed tomography images (A) Computed tomography of the head reveals traumatic subarachnoid hemorrhage (yellow arrows) and hemorrhage in the right lateral ventricle (red arrowhead). (B) Computed tomography of the abdomen reveals ascites (yellow arrowheads) without other signs of infectious lesions.

A 66-year-old man with impaired consciousness and fever was admitted to our hospital. On the day of admission, he was found falling at home and transported to us. His medical history included uncompensated alcoholic liver cirrhosis; however, he did not receive treatment. He drank alcohol daily but had no history of smoking or allergies. Physical examination revealed a blood pressure of 123/85 mmHg, pulse of 120/min, respiratory rate of 26 breaths/min, and body temperature of 38.9℃. His consciousness was disturbed and registered as E4V1M4 on the Glasgow Coma Scale. The patient presented with skin jaundice, abdominal distension, and a bump on the left temporal area. Laboratory findings showed a white blood cell (WBC) count of 18,900/µL (neutrophils: 84.0%, lymphocytes: 4.0%), hemoglobin of 9.9 g/dL, platelet count of 11.5 × 104/µL, total protein of 7.0 g/dL, albumin of 1.7 g/dL, total bilirubin of 10.3 mg/dL, direct bilirubin of 6.6 mg/dL, aspartate aminotransferase of 145 U/L, alanine aminotransferase of 58 U/L, ammonia of 47 µg/dL, C-reactive protein of 18.0 mg/dL, and prothrombin time (international normalized ratio) of 2.36 (Table 1). Two sets of blood cultures obtained upon admission revealed the growth of *E. coli*. Enhanced computed tomography (CT) of the head revealed a traumatic subarachnoid hemorrhage (Figure 1A) and abdominal CT revealed ascites and atrophy of the liver, though no signs of infectious lesions were observed (Figure 1B). An ascites puncture revealed a WBC count of 14,100/µL (neutrophils: 9,447/µL) and albumin of 0.6 g/dL (Table 1). Ascites culture revealed growth of *E. coli*.

**Table 1:** Laboratory findings on admission AST: aspartate aminotransferase, ALT: alanine aminotransferase, BUN: blood urea nitrogen, Cre: creatinine, LDH: lactate dehydrogenase,  CRP: C-reactive protein, PT-INR: prothrombin time (international normalized ratio)

	Result	Reference range
Blood		
White blood cell (/µL)	18,900	3,900-9,800
Neutrophils (%)	84	30-70
Lymphocytes (%)	4	19-61
Hemoglobin (g/dL)	9.9	13.4-17.6
Platelet (× 10^4^/µL)	11.5	13.0-37.0
Total protein (g/dL)	7.0	6.5-8.5
Albumin (g/dL)	1.7	3.9-4.9
Total bilirubin (mg/dL)	10.3	0.2-1.2
Direct bilirubin (mg/dL)	6.6	0.1-0.7
AST (U/L)	145	8-40
ALT (U/L)	58	8-40
BUN (mg/dL)	17.7	8.0-20.0
Cre (mg/dL)	1.19	0.60-1.10
LDH (U/L)	236	124-222
Glucose (mg/dL)	123	70-110
Ammonia (µg/dL)	47	19-54
CRP (mg/dL)	18.0	0.0-0.5
PT-INR	2.36	
Ascites		
White blood cell (/µL)	14,100	not determined
Neutrophils (/µL)	9,447	< 250
Total protein (g/dL)	1.5	0.1-2.0
Albumin (g/dL)	0.6	not determined (below the total protein value)
LDH (U/L)	247	< 200

The patient was diagnosed with spontaneous bacterial peritonitis caused by *E. coli*, which was believed to have caused his hepatic encephalopathy and impaired consciousness. Subarachnoid hemorrhage was also considered to be related to disturbed consciousness. He was administered intravenous ceftriaxone (2 g every 24 h), lactulose, and branched-chain amino acid infusion. The antimicrobial susceptibility test revealed that the bacterial strain was susceptible to ceftriaxone. On day 3, his level of consciousness improved to E4V4M6; however, on day 4, his level of consciousness worsened to E3V2M4. A cerebrospinal fluid examination was performed, revealing an opening pressure of 24 cmH2O, cell count of 5,093/µL (polymorphonuclear leukocytes: 4,112/µL), glucose concentration of 2 mg/dL, and protein concentration of 206 mg/dL (Table 2).

**Table 2:** Cerebrospinal fluid findings on day 4

	Result	Reference range
Total cell (/µL)	5,093	≦ 5
Monocytes (/µL)	981	≦ 5
Polymorphonuclearcytes (/µL)	4,112	0
Glucose (mg/dL)	2	45-80
Protein (mg/dL)	206	15-45
Chloride	107	118-130

**Figure 2:**
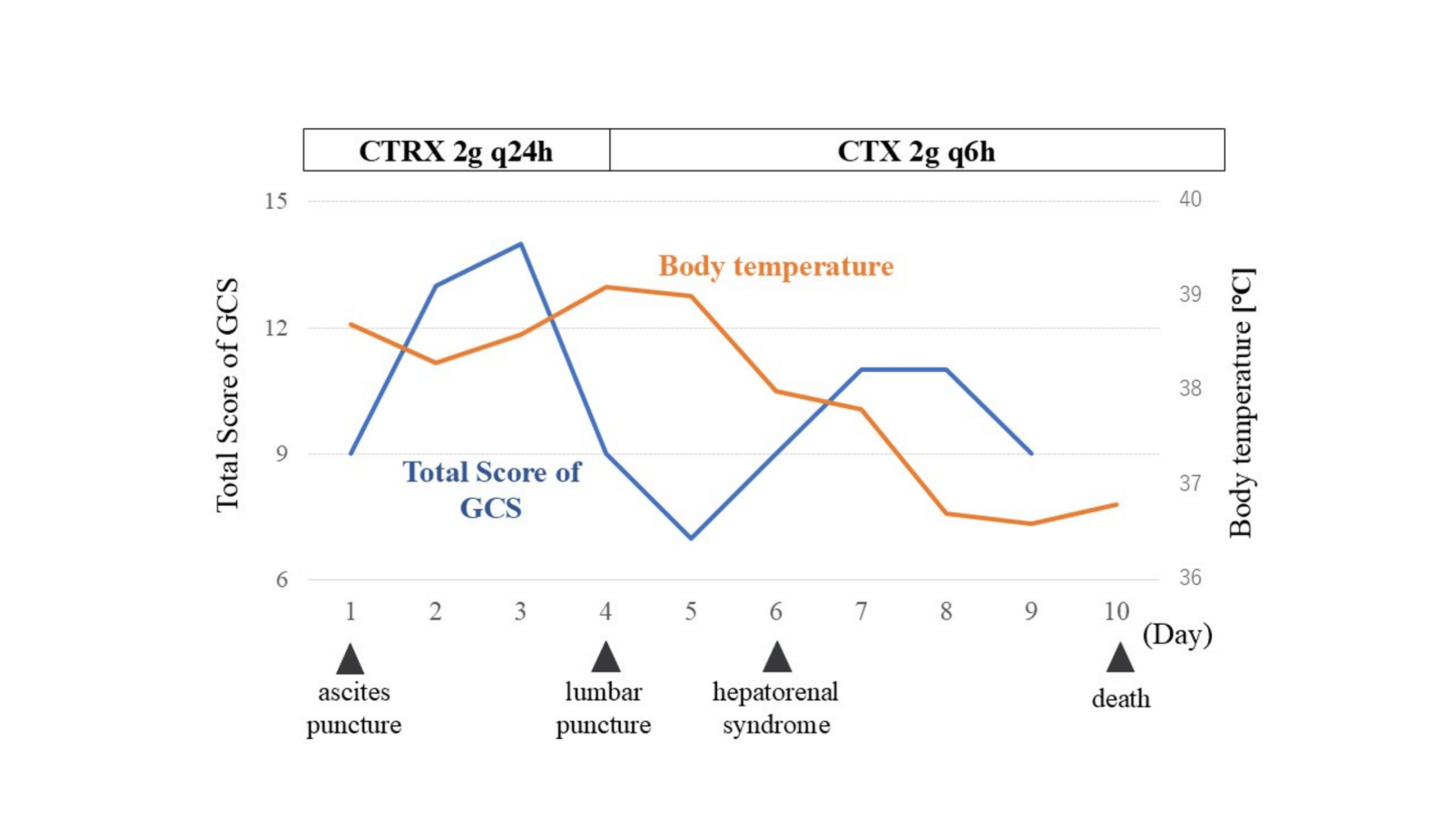
The patient’s clinical course CTRX: ceftriaxone, CTX: cefotaxime, GCS: Glasgow Coma Scale.

Gram staining of the fluid revealed gram-negative rods, and a cerebrospinal fluid culture yielded *E. coli*. The patient was diagnosed with bacterial meningitis and spontaneous bacterial peritonitis caused by *E. coli* in the absence of known risk factors. On day 4, the antibiotic was changed to cefotaxime (2 g every six hours). However, the patient developed hepatorenal syndrome on day 5 and died on day 10 (Figure 2).

## Discussion

The patient’s clinical course highlighted two important observations. First, bacterial meningitis caused by *E. coli* may occur concurrently with spontaneous bacterial peritonitis by this agent, even when no known risk factors can be identified. Second, bacterial meningitis in patients with liver cirrhosis is likely to be overlooked, which may lead to a poor prognosis.

So-called "spontaneous" bacterial meningitis in adults caused by gram-negative rods, such as *E. coli*, is rare, accounting for only 0.7-7.0% of the cases of bacterial meningitis [3,6,7]. *E. coli* is responsible for approximately 40% of cases of gram-negative rod meningitis in adults [3]. Spontaneous *E. coli *meningitis in adults frequently occurs in patients with alcoholic liver cirrhosis, diabetes mellitus, human immunodeficiency virus infection, and strongyloidiasis [4,5]. *E. coli* meningitis has a higher prevalence of coincident bacteremia and other sites of *E. coli* infection than meningitis caused by other organisms. Urinary tract infections are the most common concurrent infection [4,5]. To the best of our knowledge, there are no reports of concurrent spontaneous bacterial peritonitis and bacterial meningitis due to *E. coli*, although it could not be determined which developed first, spontaneous bacterial peritonitis or bacterial meningitis, in the current patient.

Liver cirrhosis causes neutrophil dysfunction, impairs the filtering function of the liver, and facilitates the release of endotoxins into systemic circulation via the hepatic arterial shunt [8]. The blood-brain barrier in patients with liver cirrhosis is presumed to be impaired, allowing infections to spread readily into the cerebrospinal fluid [9,10]. In a previous study of patients with liver cirrhosis, 60% (15/25) of bacterial meningitis was overlooked in the initial diagnoses, and the mean duration between arrival at the emergency room and confirmed diagnosis of bacterial meningitis was 39 h (range: 2-240 h) [11]. Bacterial meningitis in patients with liver cirrhosis has been reported to lack typical symptoms and results in a higher mortality rate (39-64%) than that in patients without cirrhosis [11-13]. The mortality rate of *E. coli* meningitis is as high as 100% in patients with Child-Pugh class C cirrhosis [4]. In the current case, the patient’s subarachnoid hemorrhage and spontaneous bacterial peritonitis prevented a prompt cerebrospinal fluid examination. Common clinical manifestations of spontaneous bacterial peritonitis include fever and abdominal pain, and approximately half of the patients present with altered mental status due to sepsis or hepatic decompensation [14].

## Conclusions

This case demonstrates that bacterial meningitis caused by *E. coli* can coexist with spontaneous bacterial peritonitis, although spontaneous *E. coli* meningitis is rare in adults. A high mortality rate has been reported for patients with liver cirrhosis who develop bacterial meningitis. Coexisting spontaneous bacterial peritonitis may obscure the symptoms of bacterial meningitis, delay the diagnosis, and worsen the prognosis. When the level of consciousness does not improve despite treatment for spontaneous bacterial peritonitis in patients with liver cirrhosis, physicians should consider possible complications of bacterial meningitis caused by the same pathogen and conduct a cerebrospinal fluid examination.
